# Chlorapatite Derived from Fish Scales

**DOI:** 10.3390/ma13051129

**Published:** 2020-03-03

**Authors:** Luyara de Almeida Cavalcante, Laís Sibaldo Ribeiro, Mitsuo Lopes Takeno, Pedro Tupa Pandava Aum, Yanne Katiussy Pereira Gurgel Aum, Jean Carlos Silva Andrade

**Affiliations:** 1 Department of Chemistry, Environment and Food; Federal Institute of Education, Science and Technology of Amazonas, Manaus 69080900, Brazil; 2 Department of Chemical Engineering, Federal University of Rio Grande do Norte, Natal 59078-970, Brazil; lais-sibaldo@hotmail.com; 3 Federal Institute of Education, Science and Technology of Amazonas, Manaus 69080900, Brazil; mitsuolopestakeno@gmail.com; 4 Faculty of Engineering, Federal University of Pará, Salinópolis, 68721000, Brazil; pedroaum@ufpa.br; 5 Department of Chemical Engineering, Federal University of Amazonas, Manaus 69080900, Brazil; yanne@ufam.edu.br; 6 Department of Materials Engineering, Federal University of Amazonas, Manaus 69080900, Brazil; jean.engmateriais@gmail.com

**Keywords:** *Arapaima gigas*, natural resource, phosphate, NaCaPO_4_, luminescence

## Abstract

The present work demonstrates the production of chlorapatite (ClAp) through thermal decomposition of chemically treated fish scales, originating from an Amazon fish species (*Arapaima gigas*). The scales were treated with hydrochloric acid (HCl) solution for deproteinization. Afterwards, the solution was neutralized by sodium hydroxide (NaOH) treatment to obtain an apatite-rich slurry. The heat treatment was carried out at different temperatures including 600 °C, 800 °C, and 1000 °C. The powders obtained were characterized through X-ray diffraction (XRD), Fourier transform infrared spectroscopy (FTIR), energy-dispersive X-ray spectroscopy (EDS), and scanning electron microscopy (SEM). The XRD analysis and FTIR spectra confirmed the incorporation of chlorine into the apatite structure. The FTIR results showed absorption bands relative to the OH^–^, PO_4_^3−^ functional groups which are a characteristic of chlorapatite. Moreover, the intensity of the OH–Cl elongation could be observed. Chlorapatite Ca_5_(PO_4_)_3_Cl, NaCl, and NaCaPO_4_ phases were identified, achieving up to 87.4 wt% for ClAp. The SEM observations show that with increasing temperature, the ClAp obtained consists of slightly larger, more crystalline grains. Furthermore, the grains ranged in size, between 1-5 μm and ClAp1000 sample recorded crystallinity of 84.27%. ClAp and NaCaPO_4_ can be used in electronics as phosphor materials due to their luminescence and biomedical applications.

## 1. Introduction

Calcium phosphate-based materials have been employed in a wide range of applications such as: biomedical [1,2,3,4]; wastewater treatment [5,6]; soil remediation [7]; foam [8]; display and solid state lightning [9]; and heterogeneous catalysis in chemical, material and industrial industries [10]. Calcium phosphate compounds can either be produced from inorganic precursors or from natural organic based materials. The use of wastes or by-products to obtain such compounds has attracted attention. Several studies have been developed using animal bones [11,12], fish scales [13], eggshells [14] and seashells [15].

Fish scales are formed by an organic component (collagen) and a mineral component (hydroxyapatite, HAp). HAp (Ca_10_(PO_4_)_6_(OH)_2_) can be extracted from fish scales through a thermal process [16], chemical treatment [17], or both [18]. Nowadays, research has been conducted towards the modification of HAp by incorporating chemical species into the apatite structure in order to improve chemical and physical properties. 

Kannan et al. [19] investigated the synthesis of chlorine substituted hydroxyapatites by aqueous precipitation. The substituted apatites show high thermal stability. Piccirillo et al. [20] carried out a combined washing-annealing process using fish scales to obtain chlorapatite (Ca_10_(PO_4_)_6_Cl_2_, ClAp), whereby the replacement of hydroxyl groups occurred, replaced with chlorine, due to the presence of NaCl in sardine scales. ClAp can therefore be used in luminescent device applications [21] as well as in biomedicine to improve resorption, mechanical properties and bioactivity [22].

Recently, orthophosphates have been widely studied with findings suggesting they are efficient luminescent host materials due to their excellent thermal and charge stabilities [23,24,25]. NaCaPO_4_, an alkali-alkaline-earth orthophosphate compound, has been reported to be a host material for potential applications in white light emitting diodes (w-LEDs), or, in other words, the next generation of solid-state light. NaCaPO_4_ exhibits strong UV absorption and higher physical and chemical stability [26]. Furthermore, the preparation of NaCaPO_4_ doped with rare-earth ions provides a strong emission intensity [27,28].

In the work presented, fish scales from an Amazon fish species (Arapaima gigas) were used to produce phosphate-based compounds through a combined chemical-calcination process. To our knowledge, this is the first recorded time fish scales have been used as a source of HAp in a reaction with a chlorinating agent to produce ClAp. The materials obtained were characterized by several techniques (X-ray Diffraction, Fourier Transform Infrared Spectroscopy, Energy Dispersive X-ray Spectroscopy and Scanning Electron Microscopy) to determine their composition and microstructural features.

## 2. Materials and Methods 

### 2.1. Synthesis of Chlorapatite Powder from Fish Scale

Fish scales (FS) from freshwater fish (*Arapaima gigas*) were obtained from a local market located in Manaus, Amazonas, Brazil. 

**Figure 1:**
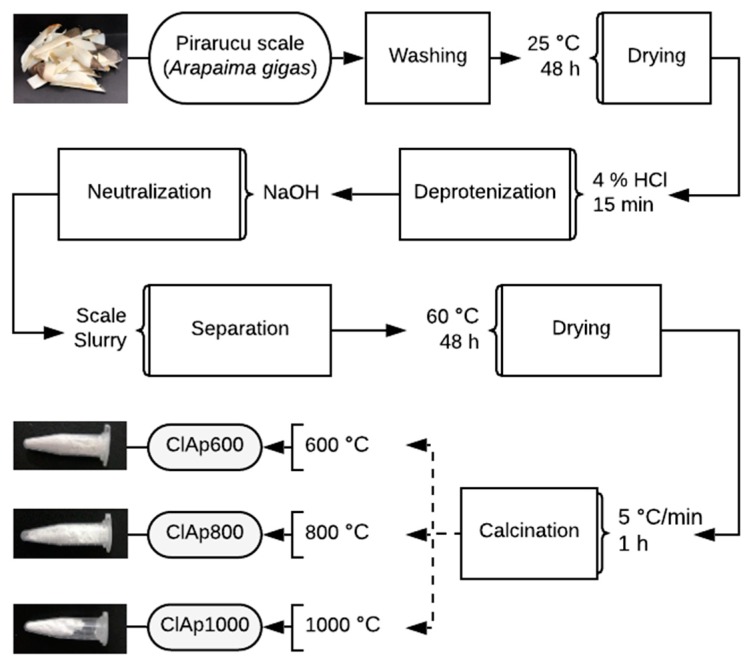
Flow chart for the processing of *Arapaima gigas* fish scales to chlorapatite (ClAp).

Figure 1 shows the flow chart for the processing of fish scales to ClAp. The scales were isolated from the fish and washed thoroughly firstly with tap water and then with distilled water to ensure the removal of undesired debris. The adhering tissue was manually scraped. After washing, the scales were dried out at room temperature. Thereafter, the scales were soaked and stirred in 4 wt% hydrochloric acid (HCl) solution for 15 min at room temperature to achieve deproteinization. Afterwards, the solution was neutralized by sodium hydroxide (NaOH) treatment to obtain apatite-rich slurry and was subsequently filtered through the process of vacuum filtration. In order to produce biogenic ClAp powder, the resulting cake was subjected to calcination at 600 °C, 800 °C and 1000 °C, at a heating rate of 5 °C /min for 1h.

### 2.2. Fish Scale and Chlorapatite Powder Characterizations

Thermal behavior of the FS was analyzed from room temperature (~30 °C) to 1000 °C with a heating rate of 5 °C/min in a nitrogen atmosphere, using a simultaneous thermal analyzer (SDT Q600, TA Instruments, New Castle, USA. The functional groups in the fish scales and synthesized powders were identified using FTIR spectroscopy (Spectrum Shimadzu, model Prestige 21, Kyoto, Japan) in the range of 400–4000 cm^−1^. The XRD analysis of the synthesized powders was performed using Bruker D2 PHASER diffractometer (Bruker AXS, Karlsruhe, Germany) with Cu Kα1 radiation (λ = 15,406 Ǻ - 30 kV - 10 mA). The scanning was done between 10 and 60° (2θ) with a 0.02° step size at 60 s each step. The crystalline phase composition of powders was analyzed with XPert HighScore Plus Software (3.0, PANalytical B. V., Almelo, The Netherlands) and Inorganic Crystal Structure Database(ICSD). In order to obtain the lattice parameters, the structural refinement was performed by the Rietveld method using the General Structure Analysis System(GSAS). The fraction of crystallinity ($x_{c}$) was estimated using Equation (1) [29].

(1) $$x_{c}=100\;\times \;\left[1-\left(\frac{I_{300}-V_{112/300}}{I_{300}}\right)\right]$$

where I_300_ is the intensity of 300 diffraction peak, V_112/300_ is the intensity of the hollow between 112 and 300 diffraction peaks of ClAp. The mean crystallite size D (Å) was calculated using the Scherrer’s equation [30].

(2) $$D\left(h\;k\;l\right)=\;\frac{0.91\lambda }{\beta_{L}cos\theta }$$

where λ is the copper wavelength, $\beta_{L}$ is the integral breadths of Lorentzian part in the pseudo-voight function of the diffraction simulated line, calculated according to Reference [31]. The morphology and the elemental compositions of scaffolds were identified using scanning electron microscopy (VEGA3- TESCLAN ANALYTICS, Fuveau, França) equipped with an energy-dispersive X-ray (EDX) analyzer (Penta FET x-3 Si). The samples were coated with a thin conductive layer of gold and analyzed at 30 kV working voltage.

## 3. Results and Discussion

### 3.1. Thermo-Gravimetric Analysis

The thermogravimetric analysis was performed on the FS to evaluate thermal stability and weight loss, noting which calcination temperatures would be most appropriate for the material studied and what its yield would be.

**Figure 2:**
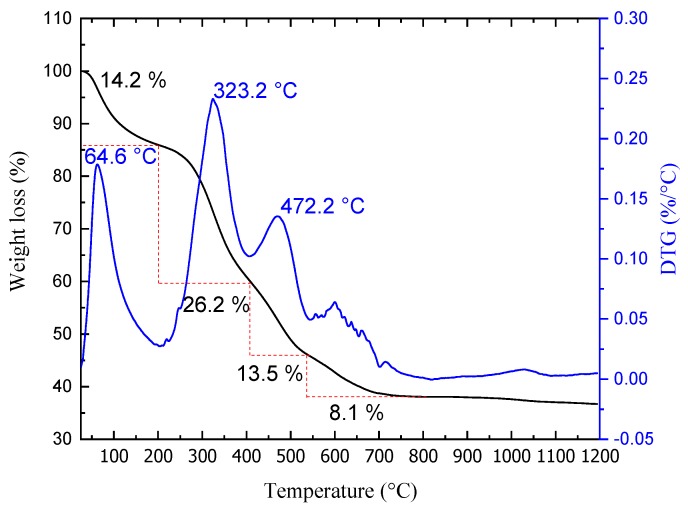
Thermogravimetric analysis.

Figure 2 shows the TGA curve and the first derivative (DTG curve; rate of change in mass) of the FS. Four different stages of weight loss can be observed in the temperature range 0–1000 °C, amounting to a total weight loss greater than 60 %. The first stage of weight loss occurs in the temperature range of 25–210 °C, reaching approximately 14.2 %. This can be understood to be related to the evaporation of the adsorbed water on the surface of the scales. The second stage, the DTG peak located at 323.2 °C refers to the degradation of organic compounds, principally collagen constituents. At this stage, the largest loss of mass occurs, at 26.2%. The first and second stages are associated with the organic molecules present in the scales: amide I, amide II and amide III, which are characteristics of type I collagen, or, in other words, the mineralized collagen (collagen fiber cross-linking) characteristic of the fish scale used [32]. In addition to the possibility of trapped water in the porous structure of the scales, there may also be a small contribution caused by the release of crystallization water in hydroxyapatite [32,33]. 

The third step relates to protein loss from organic components such as guanines, representing a total weight loss of 13.5%. The smallest loss of 8.1% can be seen between 540 °C and 820 °C. The latter may be associated with the decomposition of the inorganic phase, due to weight loss associated with the release of Na and Mg ions present in the scales of the species studied [34]; or, from decarbonization of calcium carbonate for formation of calcium oxide [35]. After 800 °C there wasn’t a significant change in weight recorded.

### 3.2. X-Ray Diffraction

**Figure 3:**
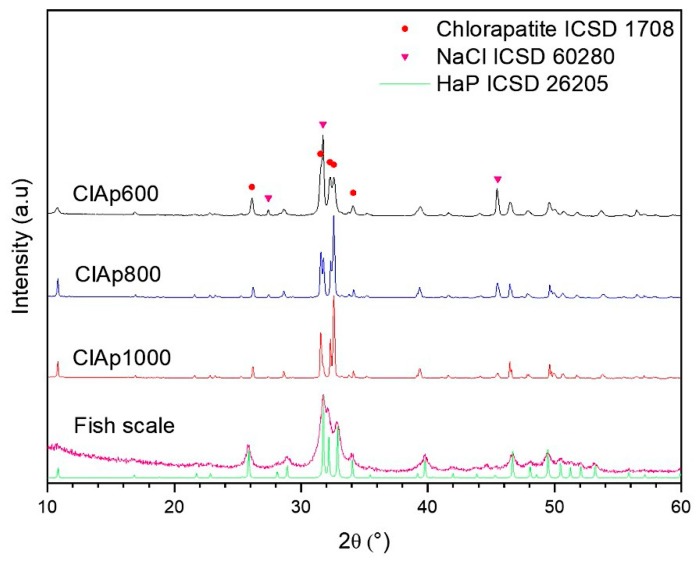
XRD patterns of the chlorapatite syntheses as a function of calcination temperature.

Figure 3 shows XRD results for the chlorapatite samples synthesized at several temperatures including 600 °C, 800 °C, and 1000 °C. Calcium phosphate (V) chloride hydroxide, ICSD card N° 1708, formed at all calcination temperatures. The chlorapatite formed has a hexagonal crystal structure, space group P63/m and chemical formula H_1.37_ Ca_9.71_ Cl_0.8_ O_25.37_ P_6._ The hexagonal phase formed at temperatures above 350 °C. Below 350 °C, its structure is monoclinic [36]. The other phase formed during chlorapatite synthesis was NaCl, ICSD card N° 60280. The formation of this phase is justified according to the chemical Equation (3), since HCl was neutralized with NaOH.

(3) $$\mathrm{HCl}\;+\;\mathrm{NaOH}\;\to \mathrm{NaCl}+H_{2}O$$

**Figure 4:**
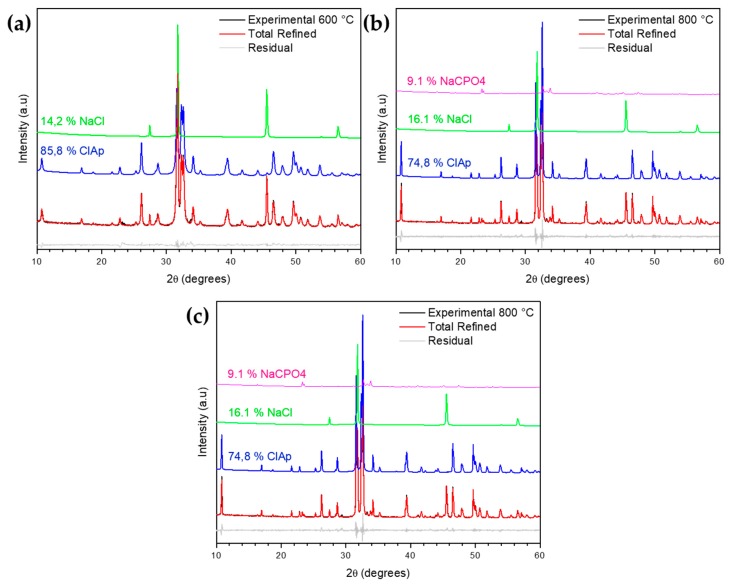
Experimental XRD pattern of the chlorapatite by the Rietveld method: (**a**) ClAp600; (**b**) ClAp800; and (**c**) ClAp1000.

The refinement shown in Figure 4a–c records the phases of chlorapatite present in all samples: ClAp600; ClAp800; and ClAp1000. The XRD pattern of the chlorapatite calcined samples (1 h of overlap) was achieved through fitting according to the Rietveld refinement method method, followed by the deconvolution of the phases in order to obtain the percentage of calculated phases. Through analyses of the calculations of percentage by weight, it was confirmed that the ClAp1000 sample presented the highest percentage of chlorapatite, at approximately 87.4%. wt.

Table 1 shows the percentage of phases found for all synthesized samples and provides the refinement and reliability parameters. The samples calcined at 800 °C and 1000 °C were observed to gain a new phase formation, namely, NaCaPO_4_ ICSD card N° 5629. It is suggested that in these calcination temperatures, the breakage occurs in apatite-linked chloride ions which, in turn, favors the binding of sodium ions.

The crystallite size for the chlorapatite phase was calculated for all the hkl plans using equation (2) and can be found in Table 1 [37,38]. This the values are represented with their standard deviations. When comparing the crystallite sizes of the calcined samples, it can be observed that the ClAp600 sample has the smallest size, at around 28.6 nm. The ClAp800 and ClAp1000 samples have a crystallite size of 78 (12) and 65 (8) respectively, showing a difference of about 13nm between the two. This difference in crystallite size is within the margin of error that was calculated. Therefore, there was no significant variation in crystallite size for the ClAp800 and ClAp1000 samples. However, all samples had an average crystallite size of less than 78 nm. The Ganjali et al. [39] study shows the effect of heat treatment on the structural characteristics of chlorapatite, with the authors noting an increase in the size of crystallite due to an increase in temperature. 

The crystallinity of the samples was calculated using equation (1). The values obtained were 33.66 wt. %, 66.77 wt.% and 84.27 wt.% for the samples ClAp00, ClAp800 and ClAp1000, respectively. Sharp peaks were observed at higher temperatures, owing to better crystallization. This increase in crystallinity values was also observed in the work of Piccirillo et al. [20] and Paul et al. [16], where hydroxyapatite was calcined at various temperatures.

### 3.3. FTIR Spectra Analysis

**Figure 5:**
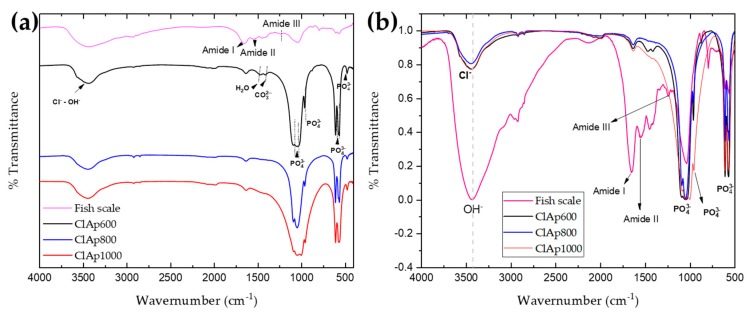
FTIR spectrum of calcined powders and FS: (**a**) Normalized spectrums; (**b**) Spectrum with real y-axis.

The formation of apatite phase in FS-derived ClAp powder was further confirmed by FTIR analysis. Figure 5a,b shows the comparative FTIR spectra of the chemically treated FS-derived powder calcined at 600 °C, 800 °C, and 1000 °C. Figure 5a shows the spectra normalized which promotes an identification comparison the identification of the bands, while Figure 5b shows the spectra with a real dimension of the *y*-axis which promotes a comparison in relation to peak intensities.

In the FTIR spectra, the bands 1654 cm^-1^, 1546 cm^-1^ and 1242 cm^-1^ were assigned to amide I, II and III of collagen [34]. The peak 1654 cm^-1^ is associated with the C=O stretching vibrations of the amide I protein; the peak 1546 cm^-1^ is associated with bending and stretching vibrations, whereby the N-H and C-N correspond to amide II; the peak 1242 cm^−1^ is associated with bending vibrations that also corresponds to amide II [16]. The inorganic components are associated with calcium phosphate formation which in turn is associated with hydroxyapatite formation, which are bands for the phosphate (562-602 cm^-1^ and 1034 cm^-1^) and carbonate anions (793 cm^-1^, 873 cm^-1^ and 1449 cm^-1^) groups [40,41].

Hence, the infrared spectra coincide with the results obtained from the X-ray patterns, with no significant changes or distortions observed in the position of functional groups and thus accounting for the formation of solid solutions in chlorine substituted apatite’s. The band of 2920 cm^-1^ refers to the stretching vibration of the C-H groups. The carbon chains of the monomers chemically constitute the scale in the peak 1636 cm^−1^ where carbonates formation can be observed [41]. The fundamental vibrational modes of (PO_4_^3−^) tetrahedra of apatite phase are witnessed in all powders in the regions of 1088 cm^-1^, 1046 cm^-1^ and 959 cm^-1.^ whereby asymmetric deformation is identified. At bands 605 cm^- 1^, 565 cm^-1^ and 473 cm^-1^, the symmetric stretching modes can be seen [42,43]. These findings are shown in Table 2.

According to Figure 5 (b), when comparing the spectra intensities of the FS sample in relation to the calcined samples there is a decrease in the intensity of the stretching vibrator observed at 3430 cm^-1^ and a reduction in the relative intensity in the band absorption of OH-, which corresponds to the formation of ClAp. The disappearance of the bands corresponding to collagen can also be noted. As the calcination temperature increases, especially in the ClAp1000 sample, there is an enlargement of phosphate bands (1034 cm^-1^) and a loss of carbon chains (2920 cm^-1^ and 1636 cm^-1^). This may indicate an approximation of the mineral state of the bone and, according to recent studies, could be indicative of good osteointegration and biocompatibility [44].

### 3.4. Powder Morphology 

**Figure 6:**
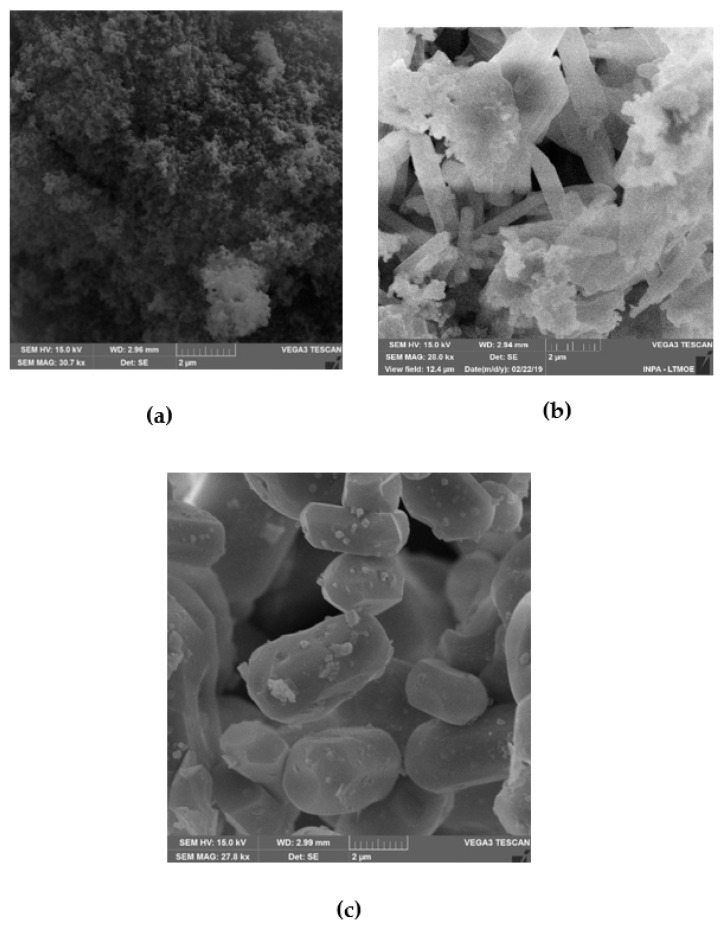
SEM images of chlorapatite calcined at different temperature: (**a**) 600 °C (ClAp600); (**b**) 800 °C (ClAp800); and (**c**) 1000 °C (ClAp1000).

Figure 6a–c shows the micrography in the samples of ClAp. The extracted ClAp shows particles with different morphologies.

In Figure 6 the SEM images show that with the increase in the calcination temperature there is an increase in chlorapatite crystals. In Figure 6a shows the ClAp600 sample, where a large number of agglomerated and ultrafine ClAp particles can be observed. In Figure 6b, the ClAp800 sample has three visible structures, one in the shape of a stick, one agglomerated and the other polygonized. On the other hand, in Figure 6c the ClAp1000 sample has a rather uniform morphology and polygonal structure. Particle size is not necessarily the crystallite size, for example, in the formation of hard agglomerates [45]. The García-Tuñón et al. [46] study shows the synthesis of ClAp with different morphologies using the molten salt method with CaCl_2_ as a flux. 

From the SEM observations, it can be noted that the ClAp obtained consists of grains with sizes smaller than 1 μm in the ClAp600 sample and sizes between 1-5 μm in the ClAp1000 sample, becoming more crystalline with increasing temperature. This same analysis shows the geometrization of the particles becoming more regular, increasing the contact area between the grains, which leads to an increase of mass transfer pathways related to volumetric diffusion phenomena [47]. This may favor the transport of cells in the microstructure of the material, such as in contact with body fluid.

Presented in Table 3, the EDS analysis confirms the presence of Ca, P and Cl. The quantitative analysis revealed that the composition ratio of calcium and phosphorous in the sample is 1.44, which is smaller than the stoichiometric ratio of Ca and P (1.67) in the pure HAp powder. This corroborates previous research, proving that the ClAp obtained is more soluble than HAp. Pure ClAp, Ca_10_(PO_4_)_6_Cl_2_, has the Ca/Cl ratio of 5 [48]. The sample ClAp1000 obtained the best Ca/Cl ratio, which suggests it was due to the increase in ClAp phase in this sample, as shown in the XRD and FTIR results, as well as the reduction in carbonates with the temperature increase.

It is worth mentioning that MEV / EDS are analyzes made on the surface of the sample, having no relation to the size of crystallites, but rather to the size of the particles/grains, which in this case are related to calcination temperatures. Therefore, the particle size does not significantly change its stoichiometric structure [45,49].

## 4. Conclusions

In the presented work, chlorapatite was synthesized by a simple thermal decomposition of chemically treated fish scales. In all of the samples, XDR analysis identified ClAp as the main component (87.4 wt%), whilst two of the samples had NaCaPO_4_ as the secondary component and all had NaCl as the third component. The degree of crystallinity and the particle size of fish scales derived ClAp increased with increasing calcination temperature. All samples recorded crystallite sizes of less than 78 nm. FTIR results show the incorporation of Cl in channel sites of the apatite. SEM has confirmed a non-homogeneous growth of particles with temperature, showing more uniform particles, with grains ranging in sizes between 1-5 μm in the ClAp1000 sample. EDS analysis has confirmed a Ca/Cl ratio of 3.84 close to the stoichiometric ClAp ratio. ClAp and NaCaPO_4_ can be used as phosphor materials in electronics on account of to their potential for luminescence. Further, chlorine-substituted HAp is a compound with an interesting potential in biomedical applications due to the fact that the presence of chlorine in the HAp lattice may improve its resorption as well as mechanical properties [22].

## Figures and Tables

**Table 1:** Volume fraction of phases and crystallite size of ClAp powder derived from fish scales at different calcination temperatures.

Sample	Calcination Temperature (°C)	ClAp (wt%)	NaCl (wt%)	NaCaPO_4_ (wt%)	Crystallite Size (nm)	R_wp_ (%)	χ^2^ (%)
**ClAp600**	600	85.8	14.2	0	28 ± 5	6.76	3.21
**ClAp800**	800	74.8	16.1	9.1	78 ± 12	7.98	4.82
**ClAp1000**	1000	87.4	6.1	6.5	65 ± 8	9.02	5.96

**Table 2:** Infrared absorption bands at ClAp600, 800, and 1000.

Absorption Region (cm^−1^)	Intensity	Designation
3400	Strong	stretch OH–Cl
2920	Weak	group stretching C–H
1636	Medium	٧1 symmetric stretching CO_3_^2−^
1088, 1046, and 959	Strong	٧3 anti-symmetric stretch PO_4_^3−^
605, 565, and 473	Strong	٧1 symmetric stretching PO_4_^3−^

**Table 3:** EDS analysis of powder samples.

Sample	Atomic %						Ca/P	Ca/Cl
	O	Ca	P	C	Cl	Na		
ClAp600	39.14 ± 0.83	5.20 ± 0.23	3.81 ± 0.18	48.00 ± 1.2	1.61 ± 0.14	2.19 ± 0.07	1.36	3.22
ClAp800	51.96 ± 8.02	8.62 ± 2.28	6.00 ± 1.69	26.26 ±10.9	3.23 ± 0.55	3.95 ± 0.54	1.44	2.67
ClAp1000	64.26 ± 3.34	14.00 ± 0.90	9.93 ± 0.44	2.90 ± 3.87	3.65 ± 1.18	4.98 ± 1.12	1.41	3.84
